# Eosinophil Lineage-Committed Progenitors as a Therapeutic Target for Asthma

**DOI:** 10.3390/cells10020412

**Published:** 2021-02-16

**Authors:** Brittany M. Salter, Xiaotian Ju, Roma Sehmi

**Affiliations:** CardioRespiratory Research Group, Department of Medicine, McMaster University, Hamilton, ON L8N 3Z5, Canada; brittmsalter@gmail.com (B.M.S.); jux@mcmaster.ca (X.J.)

**Keywords:** eosinophils, eosinophil progenitors, interleukin-5, epithelial-derived cytokines, eosinophilopoiesis

## Abstract

Eosinophilic asthma is the most prevalent phenotype of asthma. Although most asthmatics are adequately controlled by corticosteroid therapy, a subset (5–10%) remain uncontrolled with significant therapy-related side effects. This indicates the need for a consideration of alternative treatment strategies that target airway eosinophilia with corticosteroid-sparing benefits. A growing body of evidence shows that a balance between systemic differentiation and local tissue eosinophilopoietic processes driven by traffic and lung homing of bone marrow-derived hemopoietic progenitor cells (HPCs) are important components for the development of airway eosinophilia in asthma. Interleukin (IL)-5 is considered a critical and selective driver of terminal differentiation of eosinophils. Studies targeting IL-5 or IL-5R show that although mature and immature eosinophils are decreased within the airways, there is incomplete ablation, particularly within the bronchial tissue. Eotaxin is a chemoattractant for mature eosinophils and eosinophil-lineage committed progenitor cells (EoP), yet anti-CCR3 studies did not yield meaningful clinical outcomes. Recent studies highlight the role of epithelial cell-derived alarmin cytokines, IL-33 and TSLP, (Thymic stromal lymphopoietin) in progenitor cell traffic and local differentiative processes. This review provides an overview of the role of EoP in asthma and discusses findings from clinical trials with various therapeutic targets. We will show that targeting single mediators downstream of the inflammatory cascade may not fully attenuate tissue eosinophilia due to the multiplicity of factors that can promote tissue eosinophilia. Blocking lung homing and local eosinophilopoiesis through mediators upstream of this cascade may yield greater improvement in clinical outcomes.

## 1. Introduction

Asthma is a chronic airways disease clinically characterized by reversible airflow obstruction, airway hyperresponsiveness (AHR), and inflammation, affecting more than 334 million people worldwide [1,2,3,4]. Over 80% of asthma-related deaths occur in low-and lower-middle income countries, indicating that treatment and effective management of asthma saves lives. The most common phenotype of asthma is eosinophilic asthma that is effectively managed by corticosteroid therapy [1,2,3]. However, a small proportion (5–10%) of patients continue to have symptoms and persistent eosinophilia, despite high-dose oral corticosteroid therapy with substantial treatment-related side effects, and it accounts for the majority of the socio-economic and healthcare burden of asthma [4]. Therefore, exploring alternative treatment strategies is warranted. In this review, we will discuss the evidence for hematopoietic processes in eosinophilic asthma and the effect of various therapeutic targets on eosinophil levels and asthma management.

## 2. Identification and Enumeration of Hematopoietic Progenitor Cells

Under steady-state conditions, hematopoiesis primarily occurs in hematogenous sites within the bone marrow (BM), which are influenced by resident stromal cells-derived signals that maintain progenitor cells at various stages of lineage commitment. Hematopoietic progenitor cells (HPCs) are pluripotent stem cells with the capacity to self-renew and differentiate into mature blood cells. These cells are identified by surface expression of the stage-specific antigen CD34, which is a monomeric transmembrane O-sialylated glycophosphoprotein [5]. CD34 is highly expressed on primitive cells early in differentiation and progressively decreases with maturation, becoming absent on terminally differentiated cells [6]. CD34 is present on eosinophil progenitors (EoP) but not eosinophils [7]. The common myeloid progenitor gives rise to EoP, which are identified as CD34^+^ cells that co-express the interleukin (IL)-5 receptor alpha (IL-5Rα) [8,9]. The IL-5R is comprised of a unique cytokine binding α chain and common signaling β chain, which is shared with IL-3 and Granulocyte-macrophage colony-stimulating factor, also known as colony-stimulating factor (GM-CSF). The up-regulation of IL-5Rα on HPC occurs at an early stage in eosinophil development and denotes eosinophil lineage commitment [8,9,10]. Progenitor cells can be enumerated by flow cytometry or in semi-solid cultures as eosinophil/basophil colony-forming units (Eo/B-CFU) [11]. The assessment of phenotypic changes in eosinophil progenitor cells and maturing eosinophils in the bone marrow of normal subjects is detailed elsewhere [12]. The complex interplay between growth factors, cytokines, and transcription factors (TFs) controls eosinophilopoiesis, which is detailed below.

## 3. Regulation of Eosinophilopoiesis

### 3.1. Transcription Factors and Eosinophil Lineage Commitment

**Figure 1:**
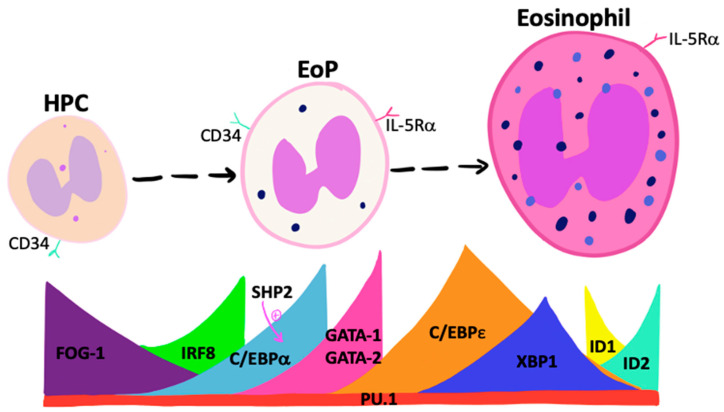
Transcription factors that instruct eosinophil differentiation. A number of transcription factors control eosinophil-lineage commitment and differentiation to mature eosinophils. A decline in FOG-1 and increased IRF8 up-regulates C/EBPα, with subsequent GATA-1/GATA-2 expression. Collective IRF8, PU.1, C/EBPα, and C/EBPε expression results in eosinophil-lineage commitment, followed by collaboration between C/EBPε, PU.1, GATA-1, and GATA-2 for progression to mature eosinophils. XBP1 and balanced ID1/ID2 expression allows for eosinophil granule protein synthesis and survival. Abbreviations: C/EBPα= CAAT/enhancer binding protein alpha; C/EBP = CAAT/enhancer binding protein epsilon; EoP = Eosinophil progenitor; FOG-1 = Friend of GATA-1; GATA-1 = Globin transcription binding factor-1; HPC = Hematopoietic progenitor cells; ID1 = Inhibitor of DNA-binding proteins 1; ID2 = Inhibitor of DNA-binding proteins 2; IL-5Rα = Interleukin-5 receptor alpha; IRF8 = Interferon regulatory factor 8.

Controlled sequential and transient expression of unique fate-determining TFs determines the lineage commitment of primitive pluripotent progenitor cells (Figure 1). TFs that promote eosinophil lineage-commitment include members of the globin transcription factor (GATA) family, where GATA-binding factor-1 (GATA-1) and GATA-binding factor-2 (GATA-2) expression are known to regulate eosinophil differentiation [13]. Studies show that although GATA-1-deficient mice have reduced fetal liver levels of EoP, GATA-2 maintains an instructive capacity comparable with GATA-1 in vitro and may compensate for GATA-1 deficiency in vivo [14]. Quantitative changes in GATA-1 expression alter HPC commitment, where moderate expression promotes eosinophil-lineage commitment while higher levels are inhibitory. Other TFs, including friend of GATA-1 (FOG-1) and interferon regulatory factor 8 (IRF8), regulate *Gata1* expression. FOG-1 is highly expressed by HPCs, acts to antagonize GATA-1, and must be down-regulated for eosinophil differentiation to proceed [15,16,17]. In contrast, loss of *IRF8* expression attenuates EoP frequency and *Gata1* expression [18].

EoP express high levels of the TF CAAT/enhancer binding protein alpha (C/EBPα) [19,20,21]. The enforced expression of C/EBPα in HPC promotes eosinophil and neutrophil development [22] and C/EBPα-deficiency in mice results in the attenuation of these cells [23]. C/EBPα expression levels impact differentiation, whereby high levels increase neutrophil development at the expense of eosinophils [24]. The specific order of GATA and C/EBPα expression is critical for eosinophil commitment [25]. Enforced expression of GATA-1 or GATA-2 in an already C/EBPα-expressing progenitor cell results in eosinophil lineage-commitment, whereas GATA-2 expression *prior* to C/EBPα leads to basophil development. Additional isoforms of the C/EBP control eosinophil developmental stages. Low levels of activator C/EBPε isoforms are expressed in HPCs and increase during IL-5-induced differentiation, whereas repressor isoforms are present during later stages of maturation [26]. Deficiency in C/EBPε results in *failed* eosinophil maturation, indicating the critical role of these isoforms in regulating eosinophilopoiesis [27].

The TF, X-box binding protein 1 (XBP1) is involved in unfolded protein responses triggered by endoplasmic reticular stress during cellular differentiation. In response to this stress, *Xbp1* mRNA is spliced by Inositol-requiring enzyme 1α (IRE1α), followed by the translation of XBP1 [28]. Accumulation of spliced *Xbp1* mRNA is only detected in precursor and not mature eosinophils. The loss of *Xbp1* expression in precursor populations results in an attenuation of eosinophil maturation and a lower frequency of EoP caused by the dysfunctional post-translational maturation of secondary granule proteins, including major basic protein and eosinophil peroxidase. Although XBP1 is not necessary for eosinophil-lineage commitment, it is an important survival factor for EoP and is critical for successful eosinophil maturation [28]. A human equivalent of this TF has not been reported to date but may provide an important therapeutic target for controlling eosinophilia.

The TF PU.1 is essential in myeloid development. The expression of *PU.1* increases gradually throughout HPC development and remains constant during terminal differentiation [29]. The disruption of *PU.1* expression causes defective granulocyte terminal differentiation, with an absence of functionally mature neutrophils and eosinophils. Gene expression analysis of PU.1-deficient fetal liver cells has shown the expression of granule proteins but little *IL-5rα* expression, suggesting that PU.1 is important but not essential for eosinophil commitment [30]. Recent studies implicate the Tribbles pseudokinase family (Trib) in controlling eosinophil-lineage commitment. Mice with germline deletion of Trib1 lack eosinophils and have greater numbers of neutrophils [24]. Trib1 expression suppresses neutrophil programing in lineage-committed EoP in response to IL-5 [31]. Trib1 regulates eosinophilopoiesis through the suppression of C/EBPα, thereby blocking neutrophil differentiation. In addition, the tyrosine phosphatase, Src homology 2 domain–containing phosphatase 2 (SHP2), stimulates *C/EBPα* expression to regulate cytokine-dependent granulopoiesis, indicating that this TF may be a critical regulator of eosinophil differentiation [32]; the blockade of SHP2 inhibits eosinophilopoiesis [33].

The inhibitor of DNA-binding proteins (ID) is a family of negative transcriptional regulators, and ID2 is up-regulated during eosinophil maturation. The ectopic expression of ID2 in human HPC results in increased mature eosinophils but no change in frequency of early precursors [34]. On the other hand, ID1, an inhibitor of terminal eosinophil maturation, declines through the differentiative process [34]. These findings suggest that ID2 enhances terminal differentiation, whereas ID1 is inhibitory, and as such, a certain ratio of expression of ID proteins is required for eosinophilopoiesis.

In summary, eosinophil lineage commitment occurs when HPC sequentially express C/EBPα, C/EBPε, IRF8, and PU.1, along with declining FOG-1 and increases in GATA-1/GATA-2 expression. Furthermore, data are suggestive of a delicate interplay between relative ratios and sequence of expression of various TFs during differentiation. Targeting specific TFs may provide effective therapeutic avenues for controlling tissue-specific eosinophilopoiesis, although this remains to be demonstrated in human disease (Figure 1).

### 3.2. Eosinophilopoietic Factors

Growth factors such as, IL-5, IL-3, and GM-CSF all participate in eosinophilopoiesis. In turn, the interaction of IL-5 and IL-5Rα expressed by EoP mediates the terminal differentiation and growth of progenitor cells, with a resultant priming of activation and survival of mature eosinophils [35]. However, IL-5-deficient mice retain homeostatic levels of eosinophils, suggesting that other factors support eosinophilopoiesis [36,37]. Additional factors, including IL-3 and GM-CSF, mediate effects via IL-3Rα and GM-CSFRα, driving early stages of differentiation, whereas the β-common chain mediates cell signaling by all three eosinophilopoietic cytokines.

More recently, epithelial-derived alarmin cytokines have been implicated in promoting eosinophil differentiation either directly or indirectly. The triad of alarmins, including IL-25, IL-33, and TSLP, is produced by airway epithelium in response to environmental and microbial stimuli. The eosinophilopoietic potential of TSLP was first demonstrated in humans using cord-derived CD34^+^ cells, where TSLP stimulated the growth of Eo/B-CFU in 14-day methylcellulose cultures, which was enhanced in the presence of IL-3 and TNFα [38]. Similarly, the outgrowth of Eo/B-CFU in cultures of progenitor cells with bronchial epithelial cell-derived supernatants from severe asthmatics was reported and shown to be attenuated by TSLP blockade [39]. Furthermore, recombinant human TSLP, at picogram levels, stimulated the formation of Eo/B-CFU, with additive effects when combined with IL-5, in vitro. At the mRNA level, a synergistic increase in GATA-2 and C/EBPα in CD34^+^ cells were detected in the presence of TSLP and IL-5, indicating that TSLP can act either alone or in concert with traditional eosinophilopoietins to promote eosinophilopoiesis [39].

Mice deficient in IL-33 and the IL-33 receptor, ST2, have reduced peripheral blood (PB) eosinophils at baseline [40,41]. The administration of exogenous IL-33 increased BM and PB eosinophils in wild-type mice, IL-33 KO but not ST2 KO mice, and this effect was inhibited by anti-IL-5 treatment [40]. Although ST2 and IL-33 KO mice have significantly reduced eosinophil numbers, BM-derived progenitor cells cultured with IL-5 from these mice differentiate into eosinophils, suggesting that IL-5-driven eosinophilopoiesis can occur in the absence of IL-33 [42]. Thus, IL-33 may promote eosinophilopoiesis indirectly by stimulating IL-5 production by either CD34^+^ cells [43] or accessory immune cells. In line with this, Boberg et al. reported that in mice challenged with house dust mite (HDM), an increase in group 2 innate lymphoid cells (ILC2) expressing ST2 was observed and these cell numbers correlated with EoP numbers [41]. The combination of HDM and IL-33 directly induced IL-5 production from BM ILC2 but not T cells to drive eosinophil differentiation. Additionally, IL-33 is postulated to up-regulate IL-5Rα expression, leading to eosinophil lineage commitment via an ST2-dependent pathway [40,42]. Other studies report that GATA-1 and GATA-2 can up-regulate ST2 expression through GATA binding sites upstream of the ST2 promoter, suggesting that IL-33 may indeed be involved in eosinophil differentiation [44]. With respect to human studies, although CD34^+^ cells express ST2, it remains unknown whether IL-33 can induce eosinophilopoiesis [43,45]. Studies have shown that the eosinophil clonogenic capacity of bronchial epithelial cell-derived supernatants from severe asthmatics is increased compared to normal and mild asthmatics, and this activity can be inhibited by TSLP but not IL-33 blockade [39]. It is possible that other airway structural cells, such as smooth muscle cells may be greater producers of IL-33 that could drive local eosinophilopoiesis within the lung, although this requires further investigation.

Lastly, with respect to IL-25, HPC and EoP express the receptor for IL-25 (IL-17RB) [46] and IL-25 can promote HPC expansion and contribution to type 2 inflammation in mice [47,48]. To date, no studies have reported either the direct or indirect effects of IL-25 on human eosinophilopoiesis, although priming effects of EoP migration have been reported [46].

With respect to cysteinyl leukotrienes (CysLT), similar to leukotriene D4 (LTD_4)_, enhances GM-CSF- and IL-5-stimulated BM Eo/B-CFU growth, which can be attenuated by CysLT1 receptor antagonism [49]. Importantly, LTD_4_ does not induce the growth of Eo/B-CFU alone but in combination with IL-5/GM-CSF. This suggests that leukotrienes behave as co-factors of eosinophilopoiesis. In addition, chemokines such as eotaxin and regulated upon activation, normal T cell expressed and presumably Secreted (RANTES), alone or in combination with IL-5, can induce eosinophil differentiation from cord blood HPC, although this was not seen in cultures of CD34^+^ cells from PB and BM of asthmatic subjects [50].

Collectively, cytokines and chemokine growth factors control lineage commitment and eosinophil maturation; further clarification is required for the tissue-specific effects of these factors and their role in homeostasis compared to disease states.

## 4. Hematopoietic Processes in Eosinophilic Asthma

The differentiation of HPC was traditionally thought to be restricted to peripheral hematogenous sites such as the BM; however, accumulating evidence suggests that progenitor cells trafficking to mucosal tissue sites of type 2 inflammation may contribute via in situ differentiative processes to local eosinophilia. Baseline cross-sectional studies initially showed that HPC (CD34^+^CD45^+^ cells) are increased in the BM and PB of atopics compared to healthy subjects [51]; the greatest number of precursor cells was detected in severe compared to mild asthmatics and non-asthmatic controls, correlating with serum IL-5 and GM-CSF levels [52]. In a baseline cross-sectional study, PB HPC from atopic or atopic asthmatics differentiated into greater numbers of Eo/B-CFU in the presence of IL-5 compared to healthy controls [51,53]. These numbers increased further in dual responder asthmatics following an asthma exacerbation [54]. The selective increase in Eo/B-CFU was also observed in asthmatic subjects undergoing stepwise withdrawal of inhaled corticosteroids until a mild exacerbation of symptoms occurred. In addition, following an inhaled-allergen (Ag) challenge, circulating Eo/B-CFU are increased 24 h post-Ag in dual responders but not early responder asthmatics [55]. Gauvreau et al. expanded on these findings to show that atopic individuals have higher HPC numbers expressing GM-CSF in blood compared to healthy controls, and atopic asthmatics challenged showed increased outgrowth of Eo/B-CFU growth with GM-CSF expression 24 h post-Ag [56]. This suggests that GM-CSF expression by HPC may provide an autocrine stimulus to enhance eosinophil differentiation. Additional studies have enumerated progenitor cells in the bronchial mucosa and reported that EoP (identified as CD34^+^IL-5Rα mRNA^+^ cells) are increased in asthmatics compared to non-asthmatic controls and that these cells correlate with the level of airway dysfunction [35].

Eosinophil-lineage committed progenitor cells identified as CD45^+^CD34^+^IL-5Rα^+^ cells by flow cytometry have been shown to be increased in the BM 24 h post-Ag challenge in mild asthmatics, with associated PB and sputum eosinophilia, and the development of AHR [8]. Similarly, Kuo et al. showed that circulating HPC express surface IL-5Rα and intracellular IL-5, with greater expression in asthmatics compared to healthy controls at baseline [57]. Thus, the concomitant expression of intracellular IL-5 and extracellular IL-5Rα on HPC indicates a potential autocrine loop that could drive eosinophilopoiesis [58]. With respect to disease severity, a 10-fold greater frequency of EoP has been reported in the sputum of severe prednisone-dependent asthmatics compared to mild asthma despite comparable numbers in PB [59]. This suggests that an exaggerated eosinophilopoietic environment may exist in the airways of eosinophilic severe asthmatics. Furthermore, EoP from severe asthmatics demonstrate a greater outgrowth of Eo/B-CFU in response to similar concentrations of IL-5 and a significantly greater spontaneous clonogenic capacity when cultured with diluent alone compared to EoP from mild asthmatics, supporting the potential for autocrine cytokine generation promoting eosinophilopoiesis in more severe disease [59]. Although not definitive, these findings support the proposal that in situ eosinophilopoiesis may contribute to the local expansion of eosinophils in the airways of severe eosinophilic asthmatics. In support of this, Cameron et al. showed that the addition of allergen or IL-5 to nasal explants from ragweed sensitive allergic rhinitics resulted in a decline in EoP numbers and concurrent increase in mature eosinophils within 6–24 h of culture which was inhibited by IL-5 blockade [60]. Similarly, another study has reported that following inhalation of IL-5, CD34^+^/IL-5Rα mRNA^+^ cells decline within the bronchial mucosa of asthmatics 24 h post-inhalation, with an associated increase in eosinophils [61], indirectly supporting the view that EoP give rise to mature eosinophils within the lung during allergic inflammatory responses in an IL-5-dependent manner.

**Figure 2:**
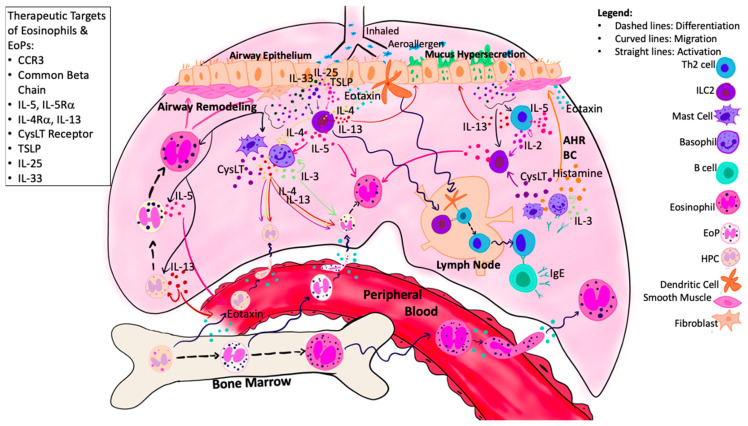
Eosinophilopoietic processes in asthma-damaged airway epithelial cells release alarmins, which activate local ILC2s, resulting in release of type 2 cytokines, including IL-5, IL-4, and IL-13. Growth factors (IL-5/IL-3/GM-CSF) produced by local effector cells and airway epithelium, in combination with alarmins, induce in situ eosinophilopoiesis. The migration of HPCs, EoPs, and eosinophils is mediated by local and systemic chemo-attractants, including eotaxin, which is enhanced by local CysLTs, alarmin cytokines, and type 2 cytokines. Within the airways, eosinophils contribute to tissue damage and airway remodeling through the production of granule proteins. Numerous targets have been identified that may attenuate both eosinophil and EoP activity, including CCR3, IL-5, IL-4/IL-13, common beta chain, CysLT receptor, and alarmins. Abbreviations: ASM = Airway smooth muscle; AHR = Airway hyperresponsiveness; BC = Bronchoconstriction; CysLT = Cysteinyl leukotrienes; Eo = Eosinophil; EoP = Eosinophil Progenitor Cell; HPCs = Hematopoietic progenitor cells; ILC2 = Group 2 innate lymphoid cells.

Accumulating evidence supports the hypothesis that following exposure to Ag, progenitor cells egress from BM and traverse to the airways where they undergo in situ eosinophilopoiesis. This process is complex and regulated by numerous mediators within both the systemic and local tissue environment (Figure 2). Moving forwards, it is important to determine the predominant mechanisms driving the eosinophilopoietic processes to develop novel targets for asthma. The trafficking of progenitor cells is further supported by increased CCR3^+^HPC numbers within the BM 24 h post-Ag challenge, which is associated with increased migrational response to eotaxin-1 (CCL11) but decreased responsiveness to tissue-retentive mediators such as stromal cell-derived factor 1α (SDF-1α), in vitro [62,63,64]. It is postulated that reduced retentivity mediated by decreased response to SDF-1 and increased response to CCL-11, mediates progenitor cell egress from the BM to the periphery during allergen-induced asthmatic responses. The effects of eotaxin-2 and eotaxin-3 were not assessed in these studies.

Schwartz et al. reported increased PB EoP in the pediatric asthma population, which negatively correlated with asthma control [65]. This suggests that a decline in PB EoPs may be associated with loss of asthma control due to the recruitment of cells into airway tissue. It remains unclear whether EoP contributes to this process simply as precursors of mature eosinophils or as a source of pro-inflammatory cytokines [43].

## 5. Targets of Airway Eosinophilopoietic Processes in Asthma

Evidence points toward local in situ hematopoiesis within the airways, leading to a significant contribution to eosinophilic inflammation. Targeting the underlying mechanisms that drive local eosinophilopoiesis poses as a therapeutic avenue to modulate airway eosinophilia. Although IL-5 has been identified as a major driver of eosinophil differentiation, many other mediators may contribute to this process. Here, we will discuss the proposed targets of eosinophilopoiesis in asthma and results from clinical trials; specific targets are summarized in Table 1.

### 5.1. Anti-Common Beta Chain Therapy

The triad of growth factors, including IL-5, IL-3, and GM-CSF, has the potential to drive eosinophilopoiesis, and as such, therapies have been developed which targt the common signaling β chain of the IL-5/IL-3/GM-CSF receptors. A study with a β chain monoclonal antibody (mAb), CSL 311, was assessed for its ability to affect eosinophilopoiesis.

With respect to in vitro experiments, CSL 311 reduced the growth of BM- and PB-derived Eo/B-CFU that were incubated with IL-5/IL-3/GM-CSF pre- and 24 h post-Ag challenge in mild asthmatics [66]. In clinical studies with asthmatics, it was shown that treatment with an anti-sense TPI ASM8 (4 mg bid and 8 mg o.d. inhaled for 4 days) directed against the β common chain and CCR3, attenuated Ag-induced airway eosinophilia [66,74] as well as CCR3^+^HPC and EoP numbers [67]. In addition, drug treatment significantly attenuated FEV_1_ during the early and late asthmatic response and it improved the MCh PC_20_ 24 post-Ag (Table 1). These findings suggest that dual blockade of the β chain and CCR3 results in a reduction of not only eosinophilopoiesis but also lung trafficking of mature and eosinophil precursor cells. As convincing as these results are, there was an incomplete ablation of airway eosinophils and EoP, underscoring the concept of additional pathways contributing to eosinophilic lung inflammation.

### 5.2. Anti-Migrational Responsiveness Therapy

The trafficking of eosinophils and progenitor cells to the lungs is a key contributing process to airway eosinophilia. Preclinical studies in mice show that treatment with a CCR3 mAb can abolish eosinophil recruitment to the lungs [75]. However, similar studies in human asthma have not yielded similar findings. Treatment with an anti-CCR3 small molecular weight antagonist (GW766994 300 mg, PO bid for 10 days) in mild–moderate asthmatics did not attenuate airway eosinophilia, EoP numbers, or cause any clinically relevant improvement in FEV_1_ [69]. A more recent study targeting CCR3 showed that pre-treatment with AXP1275 (50 mg PO o.d.) in mild asthmatics resulted in a trend toward reduced sputum eosinophils at 24 h post-Ag but no attenuation of AHR [76]. A recent study, with R321 (a CCR3 antagonist that self-assembles into nanoparticles and binds CCR3 directly), showed that treatment inhibits eosinophil lung homing and AHR in a murine model of asthma [77]. Overall, these findings raise the question as to whether additional chemo-attractants, other than the eotaxin family of cytokines (Eotaxin 1-3; CCL11, CCL24, and CCL-26, respectively) control eosinophil and EoP trafficking to the airways. A consideration from these studies is that the local eoisnophilopoietic process may not be affected by CCR3 antagonism, thus targeting processes in both trafficking to the airways and differentiation within the airways may have a more profound effect on airway eosinophilia.

### 5.3. Anti-IL-5 Therapy

Three mAbs targeting IL-5 activity have been approved for the treatment of eosinophilic asthma, including mepolizumab and reslizumab (bind directly to IL-5), and benralizumab (binds to the IL-5 and IL-5Rα). These agents cause a robust decline in PB eosinophilia, with an associated reduction in exacerbations and improved asthma control. Results from the various studies described below are summarized in Table 1.

Mepolizumab has the capacity to reduce eosinophil numbers across several body compartments, including BM, PB, and bronchial mucosa. However, the translation of reduced systemic or local airway eosinophilia into meaningful clinical outcomes varies between clinical trials [2,59,61,78,79,80,81,82,83,84,85]. Menzies-Gow et al., reported that treatment of mild asthmatics with mepolizumab 750 mg IV (1, 4, 8 weeks) resulted in significantly decreased BM and PB eosinophils (70% and 100% respectively) but no significant effect on EoP enumerated by flow cytometry or methyl-cellulose culture [70,82]. A significant but partial attenuation of EoP (detected as CD34^+^IL-5Rα mRNA^+^ cells) and mature eosinophils (52% and 55%, respectively) was detected in bronchial mucosa, indicating a partial role for IL-5 in driving eosinophilopoiesis in the lungs of mild asthmatics. Additional factors (IL-3 and GM-CSF) may contribute to driving eosinophilia in systemic hematogenous niches in these subjects. Studies in prednisone-dependent severe asthmatics (sputum eosinophils >3%) whose disease was postulated to be primarily driven by IL-5, reported that after 10 weeks of treatment with mepolizumab 100 mg subcutaneous (SC), there was a rapid and sustained reduction in PB eosinophils, with an associated increase in EoP caused by the IL-5 blockade of terminal differentiation [59]. In contrast, there was no significant treatment effect on sputum eosinophils or EoP numbers in this small group study. A proposed explanation is that in situ eosinophilopoiesis may be the predominant process contributing to airway eosinophilia in severe asthma, and that SC administration did not provide adequate airway bioavailability of mepolizumab. To understand this further, Mukherjee et al. conducted a study to compare the efficacy of weight-adjusted reslizumab 3 mg/kg IV in severe asthmatics (>3% sputum; >300 cells/µL eosinophils) previously treated with mepolizumab 100 mg SC q4 weeks for at least 1 year [71]. Two infusions of placebo q4 weeks was followed by four infusions of reslizumab 3 mg/kg q4 weeks (16 weeks total). Reslizumab attenuated sputum and PB eosinophils by 91.2% and 87.4%, respectively. Reslizumab reduced sputum and PB HPC numbers, along with PB EoP, but it had a limited effect on sputum EoP. Overall, IV reslizumab reduced systemic and local eosinophils, resulting in a clinically relevant improvement in FEV_1_ and symptom scores. Other studies report that reslizumab can reduce eosinophils across varying compartments, but progenitor cells were not outcome measures [86,87,88,89,90,91,92,93]. Nonetheless, although reslizumab looks promising for the reduction of circulating and local eosinophilia, treatment does not completely ablate mature or immature eosinophils, suggesting that additional factors, aside from IL-5, within the local airway environment can regulate in situ eosinophilopoiesis.

Clinical trials with benralizumab in mild asthma have shown that subsequent treatment results in a long-lasting reduction of eosinophils [94,95,96]. A Canadian sub-study of the phase III ZONDA trial reported that 28 weeks of treatment with benralizumab 30 mg SC q4 weeks significantly attenuated PB and sputum eosinophils, and it similarly reduced PB EoP numbers in prednisone-dependent severe eosinophilic asthmatics (>3% sputum; >300 cells/µL eosinophils) [72]. The number of IL-5-responsive PB Eo/B-CFU significantly decreased with treatment as did sputum EoP numbers, in contrast to the report with mepolizumab caused by benralizumab mediated antibody-dependent cell cytotoxicity of all IL-5Rα-expressing cells [72]. It is proposed that the effective attenuation of mature eosinophils as well as local EoP in the airways contributed to significant attenuation of sputum EoP. This was associated with a steroid sparing effect and improvement in asthma control, indicating that controlling local eoisnophilopoietic processes may be an important process by which airway eosinophils arise in severe eosinophilic asthmatics.

### 5.4. Anti-IL-4/IL-13 Therapy

IL-4 and IL-13 play a role in promoting type 2 inflammation through acting as chemo-attractants for eosinophils and progenitor cells to the airways. Studies indicate that IL-4 and IL-13 can directly prime the migrational response of progenitor cells in vitro and that epithelial-derived alarmin cytokines (TSLP, IL-33, and IL-25) prime EoP migration by stimulating the autologous production of IL-4 and/or IL-13 [45,46]. The targeting of both IL-4 and IL-13 has been postulated as a novel treatment for asthma.

Dupilumab is an mAb that binds to the IL-4 receptor α chain and prevents the binding of both IL-4 and IL-13. Moderate to severe asthmatics (>3% sputum; >300 cells/µL eosinophils) treated with dupilumab 300 mg SC once weekly for 12 weeks had a significant reduction in exacerbations, serum eotaxin, and TARC levels, but no clear pattern of change in PB eosinophils [97]. Other studies have shown that dupilumab treatment causes a transient increase in PB eosinophils compared to placebo or corticosteroid treatment [98,99]. These findings suggest that the blockade of IL-4/IL-13 reduces eosinophil migration to local tissues, resulting in transient increases of PB eosinophils.

With respect to IL-13-targeted therapy, the treatment of uncontrolled asthmatics with the mAb lebrikizumab 37.5 mg or 125 mg SC q4 weeks for 52 weeks resulted in increased peripheral eosinophilia due to reduced migration to the airways [100,101]. Subsequent phase III trials did not show any reduction in asthma exacerbation; thus, the use of anti-IL-13 agents has lost favor [101]. An explanation for this may be that the blockade of IL-13 alone is inadequate to achieve asthma control due to overlapping roles with IL-4.

These studies support the notion that IL-4/IL-13 signaling pathways can enhance the migration of mature and immature eosinophils and induce lung structural cells to produce the eotaxin family of cytokines. The blockade of IL-4/IL-13 results in attenuated lung trafficking of eosinophils and possibly EoP, although this has not been investigated to date. Further studies are required to better understand how the blockade of IL-4/IL-13 can affect local eosinophilopoietic processes and migration by assessing EoP numbers in the airways of patients receiving Dupilumab therapy.

### 5.5. Anti-CysLT and PPAR Agonist Therapy

CysLTs are important mediators that enhance eosinophil chemotaxis and survival as well as influence eosinophilopoiesis. Given that CysLTs can modulate both mature and immature eosinophil activity, several studies have looked at the efficacy of receptor blocking agents to treat asthma. In vitro stimulation with LTD_4_ can enhance Eo/B-CFU in the presence of IL-5 and GM-CSF, which can be reversed with montelukast [49]. Mild asthmatics treated with pranlukast 300 mg PO bid for 2 weeks resulted in reduced sputum eosinophil counts pre- and 24 h post-Ag challenge compared to placebo [73]. Similar trends were seen for eotaxin and IL-5 levels. Pranlukast also significantly attenuated Ag-induced increases in IL-5-responsive Eo/B-CFU in BM and sputum CCR3^+^HPC numbers (Table 1). These findings suggest that CysLT receptor antagonists decrease airway eosinophilia through blocking the migration of mature and immature eosinophils from the BM to the airways, and the eosinophilopoietic potential of progenitor cells.

Peroxisome proliferator-activated receptor (PPAR) agonists have been proposed as novel agents to reduce airway inflammation. Murine models of asthma have shown that PPAR agonists can inhibit airway eosinophilia and AHR [102]. With respect to human studies, Smith et al. demonstrated that in vitro treatment with rosiglitazone (PPAR gamma agonist) reduced Eo/B-CFU growth in the presence of IL-5 and IL-3 [103]. In 2017, a clinical trial assessed the effect of pioglitazone on prednisone-dependent severe asthma [104]. Patients were treated with pigolitazone 30 mg PO daily for 2 weeks, followed by 45 mg daily for 14 weeks. No difference was found regarding peripheral eosinophil counts or lung function and 14% of subjects experienced severe adverse events, resulting in drug discontinuation.

### 5.6. Anti-Alarmin Therapy

Alarmins have emerged as novel mediators of eosinophilic inflammation through directly activating ILC2 and CD4^+^ T cells to produce type 2 cytokines. Alarmins can also directly or indirectly affect EoP activity, migration, and differentiation. Unfortunately, few studies have investigated the effect of blocking these cytokines on eosinophilopoietic processes in human asthma.

Initial animal studies have shown that TSLP blockade can inhibit Ag-induced type 2 inflammation, airway eosinophilia, and AHR [105,106,107]. Our own in vitro findings demonstrate that TSLP-mediated eosinophilopoietic activity in bronchial epithelial cell supernatants from severe asthmatics and that TSLP, alone, can stimulate Eo/B-CFU formation in 14-day methycellulose cultures enhanced by IL-5 [39]. Gauvreau et al. reported that treatment with an anti-TSLP mAb, AMG 157, 700 mg IV q4 weeks for 12 weeks in mild asthma yielded a significant reduction in PB and sputum eosinophils at baseline and 24 h post-Ag challenge in mild asthmatics [108]. Similarly, a Phase IIb (PATHWAY) trial with tezepelumab (70 mg or 210 mg q4 weeks or 280 mg q2 weeks for 52 weeks) as add-on therapy in uncontrolled moderate-to-severe asthmatics showed persistent decreases in PB eosinophils and type 2 cytokines, associated with an improvement in annualized exacerbation rates and improvement in lung function [109]. These findings may be attributed to TSLP blockade attenuating eosinophilopoiesis; however, further detailed studies are required to assess this hypothesis.

IL-33 can induce eosinophilopoiesis and enhance EoP migration [41,45,46,47], suggesting that the blockade of the IL-33/ST2 axis may result in impaired airway eosinophilia. Murine models have shown that ST2 blockade or anti-IL-33 mAbs can reduce eosinophils and type 2 cytokines within BALF [40,110,111]. There are ongoing human clinical trials assessing the efficacy of anti-IL-33 mAbs to treat severe asthma and atopic dermatitis. A phase IIa clinical trial of etokimab 300 mg IV in atopic dermatitis demonstrated a significant reduction in symptom and severity scores, along with reduced peripheral eosinophilia 29 days post-administration [112]. A phase IIa trial with etokimab 300 mg IV in peanut allergy reported reduced type 2 cytokine levels and ST2^+^CD4^+^ T cells upon peanut-induced T cell activation compared to placebo [113]. However, this study did not report eosinophil counts. Interim analyses of a phase IIa study with etokimab 300 mg IV in severe asthmatics (>300 cells/µL eosinophils) reported improvements in FEV_1_ at day 2 compared to placebo, which remained significant at day 64 [114]. Improvements in lung function were associated with reduced PB eosinophils by 31% and 46% at days 2 and 64, respectively. The finalized results of the asthma-related clinical trials will shed light on whether blockade of the IL-33/ST2 axis can affect eosinophilopoiesis and translate into clinically relevant outcomes.

With respect to IL-25, mouse models have shown that the administration of IL-25 results in increased AHR, type 2 inflammation, and airway eosinophilia [115,116,117], which can be reversed by the blockade of IL-25 [118,119]. To our knowledge, ABM125 is the only anti-IL-25 mAb currently in preclinical development, and more time will be needed to see how this agent affects human eosinophilia.

## 6. Conclusions

We have provided evidence that systemic and local eosinophilopoiesis are important contributing processes to airway eosinophilia in asthma. There is a complex interplay of surrounding mediators that influence EoP egress from BM, trafficking to the airways, and in situ eosinophilopoiesis (Figure 2). Understanding what signaling pathways can influence EoP is key to developing therapeutic targets for the attenuation of airway eosinophilia. As promising as anti-IL-5 therapy is, there appears to be other underlying mediators independent of IL-5 that drive eosinophilopoiesis. A comparative analysis between these treatment strategies will help determine the most efficacious therapy for attenuating eosinophilic inflammation. There should be consideration for therapeutic agents that block multiple processes involved in airway eosinophilia, including mature and immature eosinophil migration, eosinophilopoiesis, and cell activation. Another novel approach may be to target ILC2, which are primarily activated by alarmin cytokines to produce copious amounts of IL-5 and IL-13. Uncontrolled ILC2 activity may be a key contribution to corticosteroid resistance in severe asthma. Thus, targeting these upstream cells may result in subsequent down-regulation in both mature and immature eosinophil activity. We propose that combined targeting of redundant pathways that control multiple functions of mature and immature eosinophils is necessary to ameliorate type 2 inflammation and create a more robust reduction in airway eosinophilia.

## Figures and Tables

**Table 1:** Therapeutic targets of both mature and immature eosinophils.

Study	Therapeutic Agent	Subjects	Effects on Mature Eosinophils	Effects on Eosinophil Progenitors	Other Inflammatory Outcomes
Pageau et al. 2011 [66]	TPI ASM8 (4 mg bid, 8 mg o.d. inhaled for 4d) Anti-sense oligonucleotide against common β chain and CCR3	MA	Reduced SP Eos	Reduced SP CCR3^+^HPC and EoP	Reduced SP ECP levels
Imaoka et al. 2011 [67]	TPI ASM8 (4 mg bid or 8 mg o.d. inhaled, for 4d) Anti-sense oligonucleotide against common β chain and CCR3	MA	Reduced SP Eos at 7 h and 24 h post-Ag	Reduced SP CCR3^+^HPC at pre- and 24 h post-Ag No effect on total HPC	Not reported
Panousis et al. 2016 [68]	CSL 311 (100 µg/mL) Common β chain mAb	HC and MA	Reduced PB Eos survival	Decreased BM and PB Eo/B-CFU pre- and 24 h post-Ag	Not reported
Neighbour et al. 2013 [69]	GW766944 (300 mg PO bid for 10d) CCR3 Antagonist	MA	No effect on PB or SP Eos	No effect on PB or SP EoP	Not reported
Menzies-Gow et al. 2003 [70]	Mepolizumab (750 mg IV at 1, 4, 8 weeks) Anti-IL-5 mAb	MA	Reduced BM and PB Eos	Reduced EoP in bronchial mucosa No effect on BM or PB EoP or Eo/B-CFU	Not reported
Sehmi et al. 2016 [59]	Mepolizumab (100 mg q4 weeks SC for 10 weeks) Anti-IL-5 mAb	SA (>3% SP Eos, >300 cells/µL)	Reduced PB Eos No effect on SP Eos	Increased PB EoP No effect on SP EoP	-Not reported
Mukherjee et al. 2018 [71]	Reslizumab (3.0 mg/kg IV q4 weeks, total of 16 weeks) Anti-IL-5 mAb	SA (>3% SP Eos, >300 cells/µL)	Reduced PB and SP Eos	Reduced PB and SP HPC, PB EoP No effect on SP EoP	Not reported
Sehmi et al. 2018 [72]	Benralizumab (30 mg SC q4 weeks for 28 weeks) Anti-IL-5Rα mAb	SA (>3% SP Eos, >300 cells/µL)	Reduced PB and SP Eos	Reduced PB EoP Trend toward decrease in SP EoP	Not reported
Parameswaran N et al. 2004 [73]	Pranlukast CysLT Receptor Antagonist (300 mg PO bid for 2 weeks)	MA	Reduced SP Eos pre- and 24 h post-Ag Reduced SP EG2+ cells pre- and 24 h post-Ag	Reduced BM Eo/B-CFU and CCR3^+^HPC	Trends for reduced SP cells + for IL-5 Eotaxin and RANTES 24 h post-Ag

Abbreviations: Ag = Allergen; bid = Twice daily; BM = Bone marrow; CysLT = Cysteinyl Leukotriene; d = Days; ECP = Eosinophil cationic protein; EoP = Eosinophil progenitors; Eos = Eosinophils; Eo/B-CFUs = Eosinophil/Basophil-colony forming units; h = Hour; HC = Healthy controls; HPC = Hematopoietic progenitor cells; MA = Mild asthmatics; mAb = Monoclonal antibody; PB = Peripheral blood; PO = oral; SA = Severe asthmatics; SC = subcutaneous; SP = sputum.
